# A systematic investigation of the invariance of resting-state network patterns: is resting-state fMRI ready for pre-surgical planning?

**DOI:** 10.3389/fnhum.2013.00095

**Published:** 2013-03-26

**Authors:** K. Kollndorfer, F. Ph. S. Fischmeister, G. Kasprian, D. Prayer, V. Schöpf

**Affiliations:** ^1^Department of Radiology, Division of Neuro- and Musculoskeletal Radiology, Medical University of ViennaVienna, Austria; ^2^Department of Neurology, Study Group Clinical fMRI, Medical University of ViennaVienna, Austria

**Keywords:** functional connectivity, resting-state network (RSN), resting-state, fMRI, default mode network, reproducibility

## Abstract

**Objectives:** Measurements of resting-state networks (RSNs) have been used to investigate a wide range of diseases, such as dementia or epilepsy. This raises the question whether this method could also serve as a pre-surgical planning tool. Generating reliable functional connectivity patterns is of crucial importance, particularly for pre-surgical planning, as these patterns may directly affect the outcome.

**Methods:** This study investigated the reproducibility of four commonly used resting-state conditions: fixation of a black crosshair on a white screen; fixation of the center of a black screen; eyes-closed and fixation of the words “Entspann dich!” (Engl., “relax”). Ten healthy, right-handed male subjects (mean age, 25 years; SD 2) participated in the experiment. The spatial overlap for different RSNs across the four conditions was calculated.

**Results:** The spatial overlap across all four conditions was calculated for each seed region on a single subject and at the group level. Activation maps at the single-subject and group levels were highly stable, especially for the reading network (RNW). The lowest consistency measures were found for the visual network (VIN). At the single-subject level spatial overlap values ranged from 0.31 (VIN) to 0.45 (RNW).

**Conclusion:** These findings suggest that RSN measurements are a reliable tool to assess language-related networks in clinical settings. Generally, resting-state conditions showed comparable activation patterns, therefore no specific conditions appears to be preferable.

## Introduction

The possibility of performing functional magnetic resonance imaging (fMRI) without stimulation, as an easy way to obtain insight into the spatiotemporal distribution of resting-state networks (RSNs), has revolutionized neuroscience research. It has been demonstrated that RSNs are organized as specific functional networks across the brain, demonstrating characteristic spatial and temporal changes independent of condition (sleep, task performance, rest, anesthesia) or age (fetuses, preterms, infants, adults) (see, e.g., Schöpf et al., 2012a,b). In particular, in patient groups that show a lack of task cooperation, as, for example, in patients with neurodegenerative or neuropsychiatric diseases (Auer, 2008), resting-state fMRI has become quite popular as a method by which to gain new insights into these diseases (Fox and Greicius, 2010). RSNs are typically characterized by spontaneous low-frequency fluctuations (<0.1 Hz) and are observed throughout the whole brain.

In several previous studies, state-dependent differences were observed in the functional connectivity of resting-state networks (fcRSN) (Fransson, 2006; Newton et al., 2007; Bianciardi et al., 2009; Yan et al., 2009; Van Dijk et al., 2010). Some of these studies (Yan et al., 2009; Van Dijk et al., 2010) compared the differences between frequently presented resting-state conditions: eyes-open with fixation; eyes-open without fixation; and eyes-closed. Independent of the resting-state condition, subjects or patients are usually instructed to relax, not to think of anything in particular, and not to fall asleep during the scanning session. These studies have shown that the eyes-open conditions evoked higher functional connectivity of the default mode network (DMN) than the eyes-closed condition. Apart from lower-vigilance states, such as mind wandering, day dreaming, or musing about the recent past (Mason et al., 2007; Buckner et al., 2008), the DMN is associated with monitoring the functions of sensory input (Gilbert et al., 2007; Hahn et al., 2007). Therefore, activities within the DMN seem to be attenuated when the eyes are closed. However, Fox et al. (2005) found no differences in the DMN between resting-state conditions for the eyes-closed condition, the eyes-open without fixation in low-level illumination condition, and the eyes-open condition with fixation of a crosshair.

A recently published study on the interpretation of deactivations in neuroimaging studies (Hayes and Huxtable, 2012) suggested resting-state fMRI measurements before and after task- or stimulus-related fMRI experiments would enable better interpretation of activity in the task itself. In this context, the stability of RSN patterns, independent of the resting-state condition, is of major importance.

The variations in the results of fcRSN measurements raise questions concerning the reproducibility, accuracy, and specificity of resting-state fMRI not only for basic neuroscience research. Resting-state connectivity measures are also proposed as a promising practical tool in a wide range of clinical applications, especially for pre-surgical planning (e.g., epilepsy) (see, e.g., Lui et al., 2008; Bettus et al., 2009). Hence, generating reliable functional connectivity measurements is of particular importance. The reproducibility of functional connectivity measurements within one subject and across subjects has been discussed critically. A recent study (Chou et al., 2012) obtained high reproducibility within one subject, but substantial variation in fcRSNs between subjects. However, other investigations found a high degree of reproducibility for functional connectivity in the DMN (Meindl et al., 2010) and in the motor network (Amann et al., 2009) across subjects, as well as a significant correspondence between different statistical methods (Rosazza et al., 2012). In most clinical resting-state studies, factors like differences in disease duration, incidence of precipitating factors, cognitive dysfunction, and surgical outcome, are considered, but the rest-task itself and how patients were instructed to perform in the resting-state has been given less attention.

In this study, we investigated the reproducibility of RSNs in a homogenous group of 10 healthy subjects, who underwent four different resting-state sessions, while factors that could potentially influence reproducibility were held constant. These four resting-state conditions have been commonly used in previous studies: (1) crosshair (rest_cross): participants had to fixate on a black crosshair on a white screen; (2) black screen (rest_black): subjects were instructed to focus on the center of a black screen; (3) eyes-closed (rest_eyes_closed): participants had to rest with their eyes-closed; and finally (4) subjects were asked to fixate on the presented words “Entspann dich!” (Engl., “relax”) written in black letters on a white screen (rest_relax).

The prospective use of resting-state fMRI measures in a clinical set-up for the purpose of enabling pre-surgical planning, it is of crucial importance that the designs generate a stable and reliable signal. As fMRI reproducibility characteristics can be strongly dependent on the chosen paradigm designs (Bennett and Miller, 2010), it is important to formally examine reproducibility measures for specific conditions. Therefore, we analyzed differences in functional connectivity patterns induced by the four resting-state conditions (1–4) in distinct networks: the DMN; the visual network (VIN); the sensorimotor network (SMN); the reading network (RNW); and the auditory network (AUD).

## Materials and methods

### Subjects

Ten healthy, right-handed male subjects (mean age, 25 years; SD 2) were included in the study. All participants had normal or corrected-to-normal vision. All subjects were informed about the aim of the study and gave their written, informed consent prior to inclusion. The study was approved by the Ethics Committee of the Medical University of Vienna. Measurements were performed at approximately the same time of the day, between 5:00 and 9:00 p.m.

### Imaging methods

Measurements were performed on a 3 Tesla TIM Trio system (Siemens Medical Solution, Erlangen, Germany) using single-shot gradient-recalled echo-planar imaging (EPI). Twenty slices (1 mm gap, 4 mm thickness) with an FOV of 210 × 210 mm and TE/TR 42/2000 ms were acquired. Slices were aligned with the connection line between the anterior and posterior commissure. Each subject underwent four resting-state conditions, each lasting for 5 min. The light in the scanning room was turned off throughout the whole measurement period.

### Resting-state conditions

Across all four conditions, participants were instructed not to think of anything in particular and not to fall asleep.

1. Rest_cross: subjects were visually presented with a black crosshair on a white background image using an MR-compatible visual stimulation system (NordicNeuroLab, Bergen, NO) and were instructed to focus on the crosshair at all times.
2. Rest_black: subjects were visually presented with a blank, black screen and instructed to focus on the center.
3. Rest_closed: subjects were instructed to keep their eyes-closed during the whole run and visual presentation was turned off.
4. Rest_relax: subjects were visually presented with the words “Entspann dich!” (Engl., “relax”) in black letters centered on a white screen and instructed to focus on the words.

Eye movement and fixation of the target were monitored using an MR-compatible eye tracker (ViewPoint EyeTracker, Arrington Research, Scottsdale, AZ) at all times. The order of the runs was identical for all subjects.

### Data analysis

**Figure 1:**
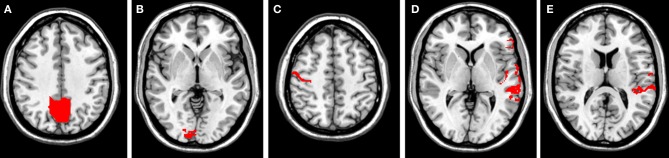
**Seed regions (red areas) for all investigated networks. (A)** Precuneus (DMN); **(B)** primary visual cortex (BA17; VIN); **(C)** right primary motor cortex (BA 4; SMN); **(D)** left reading areas (RNW); and **(E)** left auditory cortex (BA 41, BA 42; AUD).

Image preprocessing for all four runs was performed separately with SPM8 (http://www.fil.ion.ucl.ac.uk/spm/), including slice-timing and motion correction, normalization to an MNI template, and smoothing. Correlation maps were generated by computing the cross-correlation coefficient on a single-voxel basis for different regions of interest (ROIs; see Figure 1). Seed regions were chosen according to previously published literature and specified on the standard brain using the WFU PickAtlas (Maldjian et al., 2003, 2004) and processed using MarsBaR v0.43 (Brett et al., 2002). Chosen seed regions comprised the precuneus (DMN see, e.g., Cole et al., 2010), the right primary visual cortex (BA17; VIN see, e.g., Bianciardi et al., 2009), the right primary motor cortex (BA 4; SMN see, e.g., Biswal et al., 1995), the left reading areas (BA 22, BA 44, BA 45; RNW see, e.g., Koyama et al., 2010), and the left auditory cortex (BA 41, BA 42; AUD see, e.g., Cordes et al., 2000). Correlation maps were converted to *z*-values using Fisher's r-to-z transformation, as implemented in Matlab (Matlab 7.8.0, Release 2009 Mathworks Inc., Sherborn, MA, USA) to enable parametric statistical comparison. Only positive correlations were mapped for this investigation. Single-subject analysis for all four conditions (rest_cross, rest_black, rest_eyes_closed, rest_relax) was conducted for all seed regions. A within-subject ANOVA factoring the four conditions was performed separately for all seed regions, followed by T-contrasts to compare all conditions in pairs. All resulting statistical parametric maps were thresholded at *p* < 0.001 (uncorrected) using a cluster extent threshold of 10 contiguous voxels.

As a measure of consistency of the spatial patterns, an overlap of activation maps for all four conditions was calculated at the single-subject level and group level [see Equation (1)]. For comparison of consistency across all four conditions and across all possible combinations of three conditions, an overlap was calculated at the single-subject and at the group level.

The spatial overlap for each condition and each seed voxel (Rombouts et al., 1997, 1998) was calculated by

(1) $$R_{\text{OVERLAP}}(i)=n\times \;\frac{A_{\text{OVERLAP}}(i)}{\sum_{j}A_{j(i)}}$$

where *i* represents the different seed regions, *j* the different resting-state conditions, *n* the number of conditions, *A*_*j*(*i*)_ the quantity of activated voxels for seed region *i* in condition *j*, and *A*_OVERLAP_(*i*) the quantity of identical supra-threshold voxels for all conditions for seed region *i*. *A*_OVERLAP_ is a measure to describe the quantity of voxels that are activated across all conditions. *R*_OVERLAP_ ranges from 0 (no spatial overlap) to 1 (exact overlap) and can be expressed as a percentage. This measure represents the consistency of the spatial extent of the functional connectivity, independent from the way, resting-state was induced. The application of this whole brain consistency measure was motivated by the fact that we did not want to restrict our measurements to a predefined ROI (e.g., Raemaekers et al., 2007; Caceres et al., 2009).

## Results

After successfully completing all four fMRI runs, subjects were asked which of the four resting-state conditions created the greatest state of relaxation. The majority of subjects (6/10) rated rest_relax, the last condition, to be the easiest condition in which to stay relaxed during the scan. Three subjects declared the rest_eyes_closed condition, and one subject the rest_cross condition, to induce the greatest state of relaxation.

**Figure A1:**
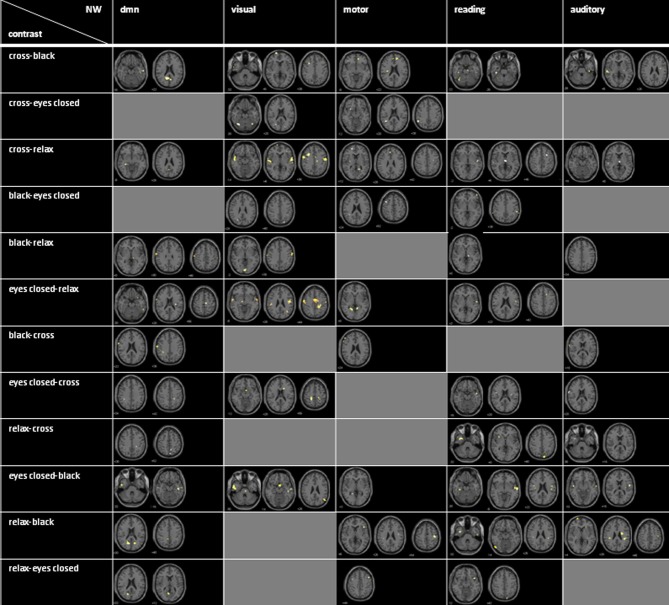
**Axial mean anatomical images overlaid by brain activation resulting from contrasts for all conditions and networks (*p* < 0.001 uncorrected for whole-brain volume analysis)**.

A complete description and visualization of all contrasts, conditions, and seed regions can be found in Figure A1 and Table A1 (both in Appendix). Results of the calculated linear T-contrast uncovered significant differences within network-specific areas for most comparisons across all resting-state conditions and all networks analyzed.

### Single-subject analysis

**Figure 2:**
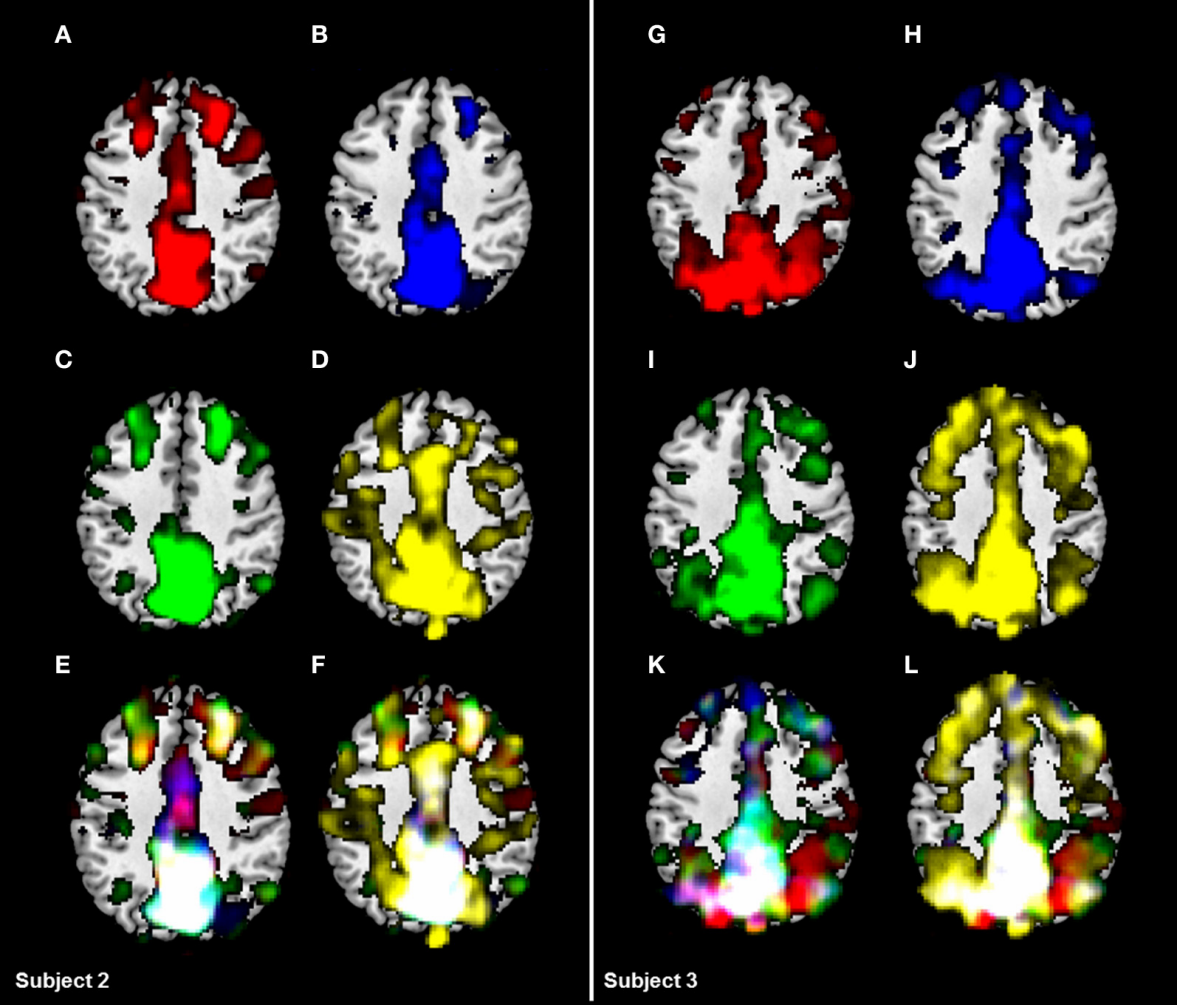
**Comparison of the DMN based on two single-subject activation maps for all four conditions. (A/G):** black screen (rest_black), **(B/H):** fixation of a crosshair (rest_cross), **(C/I):** fixation of the words “Entspann dich!” (Engl. “relax”) and **(D/J):** eyes-closed (rest_eyes_closed). Results revealed a substantial consistency across all four conditions **(F/L)** in one subject (spatial overlaps of any two conditions ranging from 0.58 to 0.66 in subject 3), but considerable differences of activation maps between two subjects for the same condition (spatial overlaps ranging from 0.39 to 0.57). However, a significant increase of overlap was found excluding the eyes-closed condition **(E/K)**.

**Figure 3:**
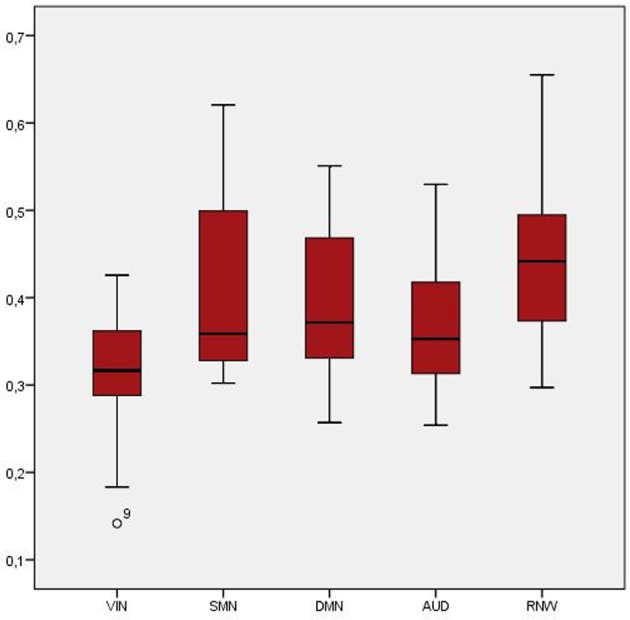
**Boxplot of overlap across all four conditions at the single-subject level**.

Results revealed a high consistency for all investigated RSN across the four conditions within one subject, but considerable differences between subjects (see Figure 2). Spatial overlaps of any two conditions within one subject ranged from 0.58 to 0.66 (exemplarily calculated for subject 3 for the DMN; for visualization see Figure 2), whereas overlaps of one condition between two subjects ranged from 0.39 to 0.57 (exemplarily calculated for subject 2 and 3 for the DMN; for visualization see Figure 2). The highest overlap across all conditions was found for the RNW (0.45), and the lowest overlap was seen in the VIN (0.31; see Table 1 and Figure 3). Results further revealed that all overlaps calculated for any combination of three conditions led to comparable spatial overlap.

**Table 1:** **Mean overlap at the single-subject level for all four conditions compared to the overlap of any possible combination of three conditions**.

**Overlap**	**DMN**	**VIN**	**AUD**	**SMN**	**RNW**
	**Mean (SD)**	**Mean (SD)**	**Mean (SD)**	**Mean (SD)**	**Mean (SD)**
All four conditions	0.39 (0.10)	0.31 (0.09)	0.37 (0.07)	0.40 (0.11)	0.45 (0.11)
Without rest_cross	0.48 (0.07)	0.37 (0.10)	0.48 (0.09)	0.50 (0.10)	0.54 (0.10)
Without rest_black	0.48 (0.12)	0.38 (0.11)	0.46 (0.11)	0.48 (0.11)	0.56 (0.10)
Without rest_eyes_closed	0.46 (0.09)	0.40 (0.09)	0.44 (0.08)	0.45 (0.10)	0.51 (0.10)
Without rest_relax	0.47 (0.10)	0.40 (0.09)	0.44 (0.10)	0.46 (0.13)	0.54 (0.13)

### Group analysis

Although group activation maps showed high consistency across all four conditions, significant variations were found in all investigated networks. For the DMN, these differences involved the precuneus, the medial temporal and frontal gyrus, the pre- and postcentral gyrus, the inferior parietal gyrus, and the paracentral lobule. Significant changes in the network-specific areas were obtained in the calcarine gyrus, the fusiform gyrus, the lingual gyrus, and the superior and medial occipital cortex. The rest_cross condition resulted in the highest functional connectivity in the right precuneus, whereas, in the resting-state condition, rest_relax functional connectivity in the precuneus was found on left side. The least precuneus functional connectivity was induced by the rest_black condition.

Significant differences in the VIN have been found across the four resting-state conditions in the calcarine gyrus, the fusiform gyrus, the lingual gyrus, and the superior occipital cortex. All of these brain areas are associated with the VIN. The rest_cross condition, compared to all other conditions (rest_black, rest_eyes_closed, rest_relax), induced the highest functional connectivity in the left lingual gyrus and the right fusiform gyrus. The rest_eyes_closed condition evoked less functional connectivity in the superior occipital cortex. Significant differences within the SMN involved areas such as the precentral gyrus, the supplementary motor area (SMA), and the postcentral gyrus. A decreased functional connectivity in the left postcentral gyrus was found in the rest_black condition. For the RNW, differences were found in the left medial temporal gyrus and the left superior temporal gyrus. Results revealed significant differences in the AUD for the superior temporal gyrus, the insular cortex, the postcentral gyrus, and the supramarginal gyrus.

The spatial consistency analysis of the group maps across resting-state conditions revealed substantial overlap in supra-threshold voxels (*p* < 0.001, FWE corrected) for all ROIs (see Table 2).

**Table 2:** **Overlap at the group level for all four conditions compared to the overlap of any possible combination of three conditions**.

**Overlap**	**DMN**	**VIN**	**AUD**	**SMN**	**RNW**
All four conditions	0.71	0.41	0.55	0.70	0.82
Without rest_cross	0.75	0.51	0.68	0.72	0.84
Without rest_black	0.79	0.43	0.70	0.77	0.84
Without rest_eyes_closed	0.76	0.41	0.54	0.75	0.87
Without rest_relax	0.74	0.62	0.54	0.73	0.86

The spatial overlap of any possible combination of three conditions was calculated for detecting one specific condition that induces most variability. The results show that all overlaps of three conditions revealed comparable overlaps, leading to the conclusion that no specific conditions appears to be preferable at group level. Therefore, no specific conditions appears to induce more variance in general.

## Discussion

fMRI is a validated and frequently used tool in pre-surgical planning for the mapping of language or motor areas (Roessler et al., 2005). Difficulties with this method may arise when patients have restricted motor or language abilities, as active participation is necessary in most fMRI paradigms. In contrast, resting-state fMRI is a method that does not require active task performance. Therefore, it is a promising tool to overcome the challenge of active participation in clinical practice. RSN measurements have experimentally been used in pre-surgical planning (Böttger et al., 2011). To date, initial experience has been gained by comparing fcRSN with functional connectivity patterns generated by a finger tapping paradigm (Zhang et al., 2009). The results of that study revealed high consistency of fcRSN patterns compared to task-based mapping of functional connectivity patterns. Less attention has been given to the impact of the resting-state condition.

In this study, we were able to demonstrate stable networks across different resting-state conditions. An analysis of the robustness of the group-mean response was performed for all resting-state conditions. Differences across the four conditions occurred in all networks investigated. However, we found that the group-level activation maps were highly stable across conditions. As previous studies have shown considerable between-subject variations (Chou et al., 2012), the analysis of consistency across the conditions was also calculated on a single-subject level. We were able to replicate these findings as single-subject overlaps for all four conditions differed across subjects within a range from 0.14 to 0.65. According to Gorgolewski et al. (2013), a reliable task may be characterized by a significantly higher within- than between-subject overlap. Results of our study revealed higher overlaps across different conditions within one subject (overlaps of any two conditions ranging from 0.58 to 0.66) than overlaps for two different subjects for the same conditions (overlaps ranging from 0.39 to 0.57).

Motion related artifacts are one of the most challenging problems in fMRI used for pre-surgical planning (Bullmore et al., 1999; Seto et al., 2001) and at very high field, as parallel imaging reconstruction artifacts, eddy currents and B0 changes due to motion increase (Beisteiner et al., 2011; Robinson et al., in revision). Known problems are head motion in slice selecting direction during one TR causing spin history effects and between image volumes causing signal changes near the ventricles and at the edge of the brain (Friston et al., 1996). Head motion artifacts are particularly severe in patient studies. Gorgolewski et al. (2013) reported stimulus related motion to be the confounding factor in explaining reliability between sessions. Therefore, resting-state fMRI could serve as a supplementary technique in pre-surgical planning not presenting with stimulus correlated motion artifacts.

In addition to the DMN, we extended our analysis to other networks, such as the VIN, the SMN, the AUD, and the RNW. These networks are of increasing importance in clinical and pre-surgical mapping studies. Thus, our results may be highly relevant for the planning and task specification of future clinical RSN trials.

The results of our single-subject analysis revealed a stable reproducibility for all investigated networks on a single-subject level, but considerable differences between subjects. These findings are in line with the results of Chou et al. (2012), who obtained high reliability of the DMN within one subject, but substantial variations across different subjects by calculating intraclass correlation coefficients (ICC) and coefficients of variance (COV). The highest overlap across all four conditions was found in the RNW (0.45), and the lowest overlap in the VIN (0.31). The poorer overlap in the VIN might have been the result of different visual input. Therefore, a spatial overlap was calculated for any possible combination of three conditions to evaluate if any condition significantly decreases variability. Exclusion of any condition led to a significant increase in spatial overlap, but no difference between any combinations of three conditions could be found. Therefore, no specific resting-state condition appears to be preferable.

A detailed discussion of our data in relation to the published literature is only possible on the topic of the DMN, as the other networks were not part of extensive investigations concerning differences across resting-state conditions. In the following paragraphs, all investigated networks will be discussed in detail.

### Default mode network (DMN)

The DMN includes parts of the medial temporal lobe, the medial prefrontal cortex, the posterior cingulated cortex, the precuneus, and the medial, lateral, and inferior parietal cortex (Buckner et al., 2008).

In this study, we found that all four resting-state conditions induced differences in functional connectivity networks within areas that are typically part of the DMN. Yan et al. (Yan et al., 2009) found differences among the three resting-state conditions of eyes-closed, eyes-open, and eyes-open with fixation. The eyes-closed condition evoked less functional connectivity, assuming that more evaluation of sensory information is needed with opened eyes, and therefore, an increased functional connectivity was found for the DMN. However, in our study, we even found differences between the three resting-state conditions with opened eyes. This variability in fcRSN activation patterns might be explained by distinctive eye movements across the four resting-state conditions (Morisita and Yagi, 2001).

For the DMN, we found a stable overlap of *R*_OVERLAP_ = 0.71 across all conditions at a group level, representing a consistent spatial extent of functional connectivity independent of resting-state condition.

### Visual network (VIN)

The VIN can be characterized by areas typically involved in processing visual stimuli, such as the mesial visual areas (e.g., striate cortex, lingual gyrus) and lateral visual areas, such as the occipital pole and the occipito-temporal regions (Rosazza and Minati, 2011). The eyes_closed condition induced less functional connectivity in the superior occipital cortex, which might be explained by the lack of visual input. These findings are in line with a study by Yang et al. (Yang et al., 2007).

The poorest overlap across the four conditions was found for the VIN. This finding could be elucidated by the use of different visually induced fcRSN conditions, which influence the functional connectivity patterns of the VIN.

### Sensorimotor network (SMN)

In the first report of a RSN (Biswal et al., 1995), spontaneous BOLD fluctuations were found in the sensorimotor cortex, the SMA, and in premotor areas during rest. In this investigation, significant differences across the four resting-state conditions were found in all areas representing the SMN. The finding of decreased functional connectivity in the rest_black condition is in line with a study by McAvoy et al. (2008), which reported a dissimilarity in networks induced by the conditions eyes-open and eyes-closed, where the first may reflect greater neuronal activity than the latter.

With an overlap of 70% across all four conditions, the SMN was found to be very robust. This high spatial reproducibility makes the SMN an interesting candidate for pre-surgical planning, e.g., to evaluate motor-related areas in patients with restricted motor abilities.

### Reading network (RNW)

Language-related areas are commonly examined for pre-surgical planning, using fMRI activation patterns during verb generation tasks, see for example (Holland et al., 2001; Eaton et al., 2008; Szaflarski et al., 2008; Tillema et al., 2008; Karunanayaka et al., 2010). Individual definition of language- and motor-related areas is of high importance, as it is widely accepted that there are no typical language or motor “centers.” These functions are rather spread across wide cortical and subcortical networks. As patients with brain tumors or epilepsy often show restricted functioning, resting-state fMRI may act as an outstanding method for pre-surgical planning.

The highest consistency across all four resting-state conditions was obtained for the RNW. With an overlap of 0.82 at the group level, the spontaneous fluctuations across all conditions was extremely stable, hence fcRSN measurements seem to be an appropriate tool for the assessment of a language network in group studies. For any 3-fold combination a minimal spatial overlap of 0.84 was found ranging from 0.84 (excluding the condition rest_cross) to 0.87 (excluding the condition rest_eyes_closed). Thus, all four resting-state conditions examined in this study appear to show robust and reliable functional connectivity in the RNW and are thus appropriate for investigations of the RNW.

### Auditory network (AUD)

The AUD involves the superior temporal gyrus, Heschl's gyrus, the insula, and the postcentral gyrus. The primary auditory cortex is known to interact with the angular gyrus, the supramarginal gyrus, Broca's area, and Wernicke's area.

Our results indicate that, among all conditions, rest_eyes_closed and rest_relax evoked the most differences concerning functional connectivity networks compared to the other conditions. With an overlap of *R*_OVERLAP_ = 0.54 in the AUD, the four resting-state conditions induced larger variability compared to the other networks, except the VIN. Yet, the underlying source of this moderate overlap remains unclear.

### Rating of relaxation

The majority of subjects rated the condition, rest_relax, as most relaxing. In this rest-task, however, less precuneus activation was found in the DMN. DMN activity is generally characterized by the absence of an active task. Without focusing attention on a task, thoughts tend to wander, participants imagine future events, or think about the recent past. In fact, participants perform an active task in the resting-state condition, rest_relax, by focusing their attention on relaxation. Subjects felt more relaxed, but mind wandering and daydreaming tendencies decreased, as the functional connectivity of the DMN is decreased compared to rest_cross or rest_black.

### Application to clinical practice

Resting-state measurements are not only of interest for basic neuroscience research. These measurements have recently been shown to allow promising insights in the epileptic brain in clinical practice (e.g., Lui et al., 2008; Negishi et al., 2011; Morgan et al., 2012).

The results of a study by Lui et al. (2008) revealed a difference in the interictal activity of the precuneus in epilepsy patients with generalized rather than partial seizures, suggesting that the lack of precuneus activation in patients with generalized seizures may contribute to the more severe interictal deficits in cognitive functions. Morgan et al. (2012) also identified resting-state fMRI as a supporting tool for pre-surgical assessment.

According to Negishi et al. (2011), RSN measurements are also an appropriate technique to predict surgical outcome in epilepsy patients. A common treatment method for intractable epilepsy is surgery, which can considerably improve epileptic symptoms. However, surgical therapy is not risk-free, and postsurgical complications can lead to cognitive dysfunction. Therefore, it is necessary to assess the possible benefit of surgical treatment. A recently published investigation by Negishi et al. (2011) showed that preoperative measurements of RSNs may serve as a predictor of surgical outcome, as patients with recurrent seizures after surgery differed in their functional connectivity compared to seizure-free patients.

Seed-based resting-state fMRI must be discussed critically in clinical practice, as mapping of a seed region may cause complications. In pathologically altered brains, an activated region may be located differently, therefore additional variance, based on different locations of the seed regions, must be recognized. Moreover, the performance of seed-based resting-state fMRI is impossible if the seed region is occupied by a tumor or a lesion.

Alterations of RSN connectivity, especially in the DMN, have been shown in neurological and neurodegenerative disorders, such as Alzheimer's disease (Auer, 2008). Initial changes can be observed even in the preclinical stages. For a reliable interpretation of the results, especially in a clinical setting, the stability of generated networks is of crucial importance, and the way the resting-state is induced should be considered. Generally, resting-state conditions with eyes-opened are preferable, as they usually evoke higher functional connectivity. According to our findings, patients who undergo fMRI for investigation of speech- and reading-related areas may also be investigated with eyes-closed conditions, considering patient comfort due to disease-related impairments. For examination of the DMN, resting-state conditions in which subjects or patients are instructed to relax by visual presentation of a word or a sentence (e.g., “relax”) should be avoided because of the lowered activity levels in the precuneus. Study protocols for fMRI resting-state measurements should be clearly defined for reliability of functional connectivity patterns and to complete information in reports. In pre-surgical planning, motion artifacts may cause considerable difficulties, as head motion artifacts are particularly severe in patients. Gorgolewski et al. (2013) identified motion as the main confounding factor in task-based fMRI. Therefore, resting-state fMRI may be a useful tool to overcome stimulus correlated motion-artifacts in pre-surgical planning.

### Limitations

A possible limitation of this study is the identical sequence of resting-state conditions, i.e., there was no randomization of the different conditions across subjects. However, it is known from several studies using the Stroop test that reading is an automatic process (see, e.g., MacLeod, 1991), which does not require the subjects' focused attention. Therefore, to prevent possible influence and carry-over effects induced by the rest_relax condition on the resting-state conditions, the run order was kept identical for all subjects.

Another limitation is eye movements, which have been suggested to possibly influence activity patterns according to Ramot et al. (2011). However, an eye-tracking device in this study was used only to verify that the subject obeyed the instructions and had his/her eyes-opened or closed. Thus, we did not record eye movements or blinking during the scanning session. Such data should be collected in further investigations to determine the exact influence of eye movements on RSNs.

## Conclusion

We were able to show that the degree of network pattern modulation induced by resting-state conditions varied across the investigated networks. The most consistent results were obtained for the RNW. Our results indicate that RSN patterns were not affected by the resting-state conditions within one subject but a considerable difference of overlap across subjects was obtained. Furthermore, we were able to show that fcRSN were highly stable at the group level, especially for the language-related network. As the overlap is comparable in any combination of three conditions, no specific condition seems to be preferable at single-subject or at group level.

### Conflict of interest statement

The authors declare that the research was conducted in the absence of any commercial or financial relationships that could be construed as a potential conflict of interest.

## Appendix

**Table A1:** **Detailed listing of all condition contrasts analyzed for all networks**.

**NW**	**DMN**	**Visual**	**Motor**	**Reading**	**Auditory**
**Contrast**					
Cross-black	r precuneus (446; 4.89)	r sup front g (100; 4.10)	l ant cingulum (68; 4.37)	r fusiform g (41; 3.93)	l fusiform g (32; 4.22)
	r med temp g (47; 3.66)	r med front g (64; 4.01)	r calcarine g (21; 3.85)	r med temp g (30; 3.70)	r sup temp g (59; 3.65)
	r med temp g (21; 3.41)	r parahippocampal g (21; 4.02)	l precentral g (26; 3.82)	r fusiform g (48; 3.70)	r med temp g (14; 3.56)
		r med temp g (25; 3.85)	r ant cingulum (20; 3.75)	l parahippocampal g (15; 3.40)	l insula (12; 3.40)
		l med front g (31; 3.44)	l sup med front g (69; 3.71)		
		r fusiform g (28; 3.42)	r insula (26; 3.66)		
		r sup temp g (32; 3.42)	r calcarine g (11; 3.64)		
		l precentral g (10; 3.40)	r cerebellum (VI) (40; 3.63)		
		r lingual g (10; 3.35)	l SMA (13; 3.51)		
			l postcentral g (19; 3.50)		
			r ant cingulum (13; 3.31)		
Cross-eyes closed		r fusiform g (106; 3.77)	r ant cingulum (18; 3.67)		
		r sup occ c (39; 3.56)	r med temp g (70; 3.63)		
		l lingual g (15; 3.53)	r inf parietal g (53; 3.50)		
		l cerebellum (43; 3.42)	l thalamus (35; 3.49)		
			r insula (26; 3.48)		
			r sup frontal g (31; 3.39)		
			r med cingulum (15; 3.39)		
			l ant cingulum (14; 3.37)		
			l sup med frontal g (12; 3.33)		
Cross-relax	r hippocampus (35; 4.03)	r precentral g (2237; 3.61)	r lingual g (20; 3.70)	l thalamus (65; 3.57)	r sup temp g (18; 3.45)
	l paracentral l (33; 3.62)	r med front g (200; 4.59)	l precuneus (70; 3.64)	l sup temp g (36; 3.51)	r thalamus (26; 3.34)
	r precuneus (18; 3.28)	l sup temp g (401; 4.56)	l med cingulum (17; 3.61)	l med frontal g (42; 3.46)	l inf front g (orb) (10; 3.26)
		r sup temp g (644; 4.42)	r sup frontal g (59; 3.49)		
		r precentral g (315; 4.25)	r ant cingulum (15; 3.48)		
		r paracentral l (465; 4.23)	l calcarine g (13; 3.46)		
		r postcentral g (55; 408)	r putamen (46; 3.34)		
		r fusiform g (28; 3.83)	l SMA (10; 3.26)		
		l inf front g (tri) (82; 3.75)			
		l insula (97; 3.70)			
		l precentral g (17; 3.67)			
		l cingulum (post) (41; 3.65)			
		l insula (31; 3.58)			
		r medial front g (65; 3.54)			
		l medial front g (19; 3.53)			
		r sup front g (27; 3.52)			
		l sup temp g (17; 3.48)			
		l sup parietal c (17; 3.82)			
		l lingual g (13; 3.37)			
		r sup temp g (48; 3.34)			
Black-eyes closed		l sup occ c (25; 3.57)	r angular g (33; 3.58)	r sup temp g (13; 3.69)	
		r sup occ c (11; 3.27)	r med front g (39; 3.41)	l supramargninal g (35; 3.69)	
			r angular g (22; 3.40)	l med front g (orb) (22; 3.45)	
				l supramarginal g (12; 3.42)	
				l inf parietal g (21; 3.81)	
				r supramarginal g (11; 3.33)	
Black-relax	r precentral g (204; 4.83)	r calcarine g (101; 4.22)		l thalamus (16; 3.78)	l precentral g (29; 3.43)
	l caudate (64; 3.69)	r precentral c (46; 3.82)			
	l inf temp g (26; 3.67)	l postcentral g (158; 3.68)			
	l paracentral l (148; 3.66)	r precentral g (10; 3.35)			
	r postcentral g (41; 3.55)				
	l postcentral g (33; 3.43)				
	l precuneus (10; 3.34)				
Eyes closed-relax	l med front g (11; 389)	r SMA (4981; 5.18)	r lingual g (149; 4.02)	g sup temp g (68; 4.09)	
	l cereb (17; 3.87)	l precentral g (963; 485)	l lingual g (52; 3.83)	l sup front g (13; 3.60)	
	r precentral g (14; 3.66)	l insula (229; 460)		r precentral g (25; 3.53)	
	r thalamus (21; 3.65)	r heschl g (902; 4.39)			
	l insula c (16; 3.61)	r thalamus (902; 4.39)			
	r paracentral l (35; 3.58)	r fusiform g (80; 4.01)			
	r thalamus (16; 3.49)	l sup temp g (37; 4.00)			
	l med temp g (16; 3.49)	l precuneus (230; 3.94)			
	l inf temp g (31; 3.39)	l angular g (63; 3.79)			
		l sup front g (97; 3.71)			
		l thalamus (14; 3.65)			
		l med front g (20; 3.61)			
		l precentral g (66; 3.56)			
		l putamen (42; 3.55)			
		r med front g (27; 3.53)			
		r inf temp g (21; 3.50)			
		r sup tem g (41; 3.49)			
		l calcarine g (14; 3.44)			
		r med temp g (12; 3.41)			
		l thalamus (32; 3.41)			
		l inf temp g (13; 3.33)			
		vermis (IV, V) (14; 3.28)			
		r rolandic op (13; 3.25)			
		r lingual g (11; 3.20)			
Black-cross	r precentral g (37; 4.19)		r inf front g (tri) (58; 3.86)		r postcentral g (59; 4.19)
	l occ med g (22; 3.76)		r inf front g (tri) (11; 3.56)		
	l thalamus (44; 3.72)				
	r postcentral g (56; 3.68)				
	r inf parietal g (25; 3.57)				
Eyes closed-cross	l med cingulum (23; 3.82)	l postcentral g (224; 4.15)		r insula (16; 3.57)	r postcentral g (49; 3.49)
	r supramarginal g (12; 3.52)	l caudate n (46; 3.91)		l med temp g (17; 3.39)	
		r paracentral l (152; 3.74)		r rolandic oper (10; 3.19)	
		r med cingulum (10; 3.60)			
		l sup temp pole (27; 3.59)			
		l med temp g (17; 3.53)			
		l thalamus (10; 3.53)			
		l angular g (21; 3.34)			
		l caudate n (14; 3.31)			
Relax-cross	l precuneus (28; 3.47)			l sup occ g (60; 4.52)	l postcentral g (15; 3.95)
	l precuneus (16; 3.39)			r med temp pole (132; 4.35)	
				l hippocampus (14; 3.54)	
				r caudate n (23; 3.54)	
				l supramarginal g (13; 3.47)	
				r lingual g (13; 3.47)	
				r insula (23; 3.46)	
Eyes closed-black	l med temp g (56; 4.38)	l rolandic oper (14; 3.60)	r lingual g (20, 3.61)	l med temp g (193; 4.69)	r sup temp g (33; 4.03)
	l med temp g (68; 3.95)	r med temp g (135; 358)		l insula (23; 4.01)	l postcentral g (63; 3.93)
	r inf temp g (53; 3.83)	r med sup temp g (11; 3.35)		l precentral g (44; 3.92)	r med front g (10; 3.74)
		r paracentral l (10; 3.26)		r rolandic oper (39; 3.81)	l rolandic oper (23; 3.68)
		l insula (10; 3.26)		r rolandic oper (23; 3.67)	r supramarginal g (31; 3.65)
				l cerebellum (crus I) (12; 3.66)	r inf occ g (14; 3.58)
				r fusiform g (64; 3.61)	r precentral g (32; 3.58)
				l sup med front g (12; 3.53)	l putamen (31; 3.54)
					l med temp (10; 3.46)
Relax-black	r cuneus (213; 4.27)		l postcentral g (126; 3.97)	l sup occ g (21; 4.38)	l supramarginal g (121; 4.50)
	r precuneus (30; 3.74)		l insula (10; 3.81)	r inf temp g (25; 3.96)	r med cingulum (39; 4.07)
	l calcarine g (97; 3.61)		l inf front g (tri) (47; 3.59)	r inf temp g (87; 3.87)	l caudate n (134; 3.85)
	l precuneus (11; 3.51)		l sup med front g (15; 3.48)	l rolandic oper (24; 3.87)	r supramarginal g (41; 3.80)
	r sup front g (10; 3.30)			l precentral g (10; 3.84)	r sup frontal g (32; 3.76)
				l cerebellum (crus I) (33; 3.73)	l inf front g (tri) (17; 3.70)
				r sup front (orb) (22; 3.73)	r sup front g (orb) (23; 3.69)
				l inf temp g (11; 3.47)	l hippocampus (20; 3.64)
				r precentral g (15; 3.30)	l fusiform g (10; 3.60)
					r inf occ g (12; 3.55)
					l post cingulum (11; 3.47)
					l caudate n (20; 3.38)
					r putamen (14; 3.35)
Relax-eyes closed	r cuneus (185; 3.99)		l med front g (85; 3.67)	l insula (29; 3.87)	
	l precuneus (15; 3.76)		r precentral g (34; 3.56)		
			l med front g (14; 3.50)		

p < 0.001 uncorrected for whole brain volume analysis. Reported are significantly activated clusters with 10 or more voxels. Clusters were automatically labeled using the AAL toolbox (Tzourio-Mazoyer et al., 2002). Numbers in brackets refer to number of significantly activated voxels and the corresponding peak Z-value.
